# Omega-3 Polyunsaturated Fatty Acid Supplementation Reduced Atrial Fibrillation Recurrence after Pulmonary Vein Antrum Isolation

**Published:** 2009-11-01

**Authors:** Dimpi Patel, Mazen Shaheen, Preeti Venkatraman, Luciana Armaganijan, Javier E Sanchez, Rodney P Horton, Luigi Di Biase, Prasant Mohanty, Robert Canby, Shane M Bailey, J David Burkhardt, G Joseph Gallinghouse, Jason D Zagrodzky, Marketa Kozeluhova, Andrea Natale

**Affiliations:** 1 Texas Cardiac Arrhythmia, St. David’s Medical Center, Austin, Texas; 2 Northeastern College of Medicine, Canton, Ohio; 3 IKEM, Czech Republic; 4 McMaster University, Hamilton, ON, Canada

**Keywords:** Polyunsaturated Fatty acids (PUFA), Atrial Fibrillation (AF), Pulmonary vein isolation (PVAI)

## Abstract

**Objective:**

To assess if patients treated with omega-3(n-3) polyunsaturated fatty acids (PUFAS) had lower procedural failure rates compared to an untreated population.

**Methods and Results:**

From January 2004 to 2007, 1500 PVAI patients underwent catheter ablation. Two hundred and eighty five (19%) patients were treated with PUFAs.  These patients were matched in a nested case controlled analysis. After matching, there were 129 patients in the PUFA group and 129 in the control group. Thirty-five (27.1%) patients in the study group had early recurrence vs. 57 (44.1%) in the control group p-value< 0.0001. Twenty-nine (23.2%) patients in the PUFA group vs. 41 (31.7%) in the non-PUFA group had procedural failure (p-value < 0.003). There were no significant differences in complications in the PUFA and non-PUFA groups.

**Conclusion:**

Patients treated with PUFAs had lower incidences of early recurrence and procedural failure compared to an untreated population.

## Introduction

Pulmonary vein antrum isolation (PVAI) is a promising treatment for drug refractory atrial fibrillation (AF). Nevertheless, some patients have unsuccessful ablations.  Therefore, any supplemental agent that may increase the likelihood of success is of particular clinical and economic interest.

Prior studies have suggested that increased fish consumption may lower blood pressure, reduce systemic inflammation, reduce postoperative AF after coronary artery bypass surgery and improve left ventricular function, all of which result in a lower incidence of AF  [1-9]. The active agent in fish oils is widely accepted to be omega-3(n-3) polyunsaturated fatty acids (PUFAs).

This study investigated if patients that underwent catheter ablation had better success rates if they were treated with PUFAs.

## Methods

### Patient Population

We screened 1,500 consecutive patients who had undergone PVAI at St David's Medical Center Austin, Texas for PVAI from January 1, 2004 to January 1, 2007. Patients were divided into those treated with PUFAs vs. untreated.  Patients were then matched in a nested case controlled methodology to limit confounders.

Patients in the study population consumed PUFAs 1 month prior to PVAI and continued PUFAs during the entire follow-up period. Since there is no evidence of a preferred PUFA dose for PVAI, we included all patients who consumed a minimum of 655 mg of fish oil capsules. The control population had not consumed PUFAs prior PVAI or during the follow-up period. Atrial fibrillation was classified according to the guidelines for catheter ablation  [10]. Early recurrence was defined as occurrences of AF during the initial 8 week interval post ablation (blanking period).  Procedural failure was defined as any recurrence of AF after 8 weeks from the time of the procedure.

The study was predominantly retrospective, however; data were collected prospectively and housed in a computerized database. The study has institutional board review permission. All patients gave consent.

### CRP levels

Baseline blood samples were obtained early in the day after an overnight fast. Plasma and serum levels were collected. Personnel blinded to clinical data performed CRP (C-reactive proteins) measurements. An ultrasensitive Enzyme Linked Immuno Sorbent Assay (ELISA) was used to measure CRP. The interassay coefficient of variation is 5.5%. Pre CRP levels were obtained no earlier than 1 month prior to the procedure. Post CRP levels were routinely collected 48 hours after the procedure.

### Ablation Protocol

#### Prior to Ablation

Antiarrhythmic drugs were discontinued four to five half-lives prior to ablation. Patients on amiodarone discontinued the medication 5 to 6 months prior to ablation. Some patients with persistent or longstanding persistent AF had a transesophageal echocardiography (TEE) or were treated with warfarin for approximately 5-6 weeks before the procedure. Warfarin was stopped 2-3 days prior to the procedure and bridged with half dose of low molecular weight heparin.

#### Ablation Procedure

All patients underwent the ablation procedure using the same ablation strategy. The details of the ablation procedure have been presented elsewhere   [11]. Briefly, our ablation strategy included PVAI guided by circular mapping catheter (Lasso-Biosense/Webster, Diamond Bar California) and intracardiac echocardiography (Acuson, Seimens, Mountain View California, Ca) along with empirical isolation of superior vena cava. In patients with paroxysmal AF, the pulmonary vein antra and a portion of the posterior wall that is contained within the area of the 4 pulmonary veins were targeted. In non-paroxysmal, the entire posterior wall extending down to the coronary sinus was targeted. In addition, lesions were also delivered on the left side of the septum. Elimination of fractionated potentials in the right atrium, left atrium and coronary sinus was performed.  Radiofrequency energy was delivered with a 3.5 mm open irrigated tip catheter (Biosense/ Webster). As a final step in all procedures, patients had an electrophysiology study with and without high dose isoproterenol challenge (20-30 mcg/min).

#### Anticoagulation

A heparin bolus (100-150U/kg) was given before transseptal punctures. The infusion rate was adjusted to keep activated clotting time (ACT) between 350-450 seconds. After PVAI, heparin was discontinued, and IV protamine 10 to 15 mg was given. Sheaths were pulled when ACT was < 280 seconds. At the end of all procedures, patients were given oral 325 mg of aspirin prior to leaving the EP laboratory. Oral anticoagulation with warfarin was resumed on the same night of the procedure. Half dose of subcutaneous low-molecular-weight heparin was administered twice a day until the international normalized ratio (INR) was ≥ 2.  Patients after June of 2005 continued their usual warfarin dose without preablation discontinuation while maintaining an INR between 2 to 3.5.

#### Follow-up

All patients were discharged on oral anticoagulation therapy (warfarin) and in most cases antiarrhythmic medications. Follow-up was scheduled at 3, 6, 9 and 12 months after the procedure and every six months henceforth. Anticoagulation and antiarrhythmics were discontinued in accordance to physician's discretion. At all the participating centres, nurses asked patients about PUFA consumption at each follow-up visit. If patients were unable to be seen, their status and PUFA consumption was assessed by a nurse practitioner via the telephone and monitoring tests were obtained by the referring physician. During the first 5 months after ablation, cardiac event monitoring was used to detect AF recurrence. Patients were asked to transmit their rhythm status three times a day and when they experienced symptoms consistent with AF.  In addition, 48- hour Holter monitoring was performed at 3, 6, 9 and 12 months and every 6 months thereafter.

### Statistical Analysis

The data from the case controlled control and study population were analyzed using SAS (version 9.2, Cary, NC). Patients were matched for type of AF, age, gender, left atrial size, ejection fraction, diabetes type II, hypertension, coronary artery disease, and stroke. Univariate analysis was performed and the mean ±  standard deviation was reported for the continuous variables. Frequency analysis was conducted for the categorical variables and goodness of fit was performed to test if the frequency distribution fits the expected distribution. Student's t-test was used to compare the means across the populations. Recurrence-free survival over time was calculated by Kaplan-Meier method. P-value of less than 0.05 was considered statistically significant.

## Results

### Patient Characteristics

Two hundred and eighty-five out of 1,500 patients that underwent catheter ablation were treated with PUFAs (19%). One hundred and twenty-nine PUFA patients and 129 non-PUFA patients were matched for the control group. The case-controlled PUFA group had lower baseline and postablation CRP levels.

**Figure 1:**
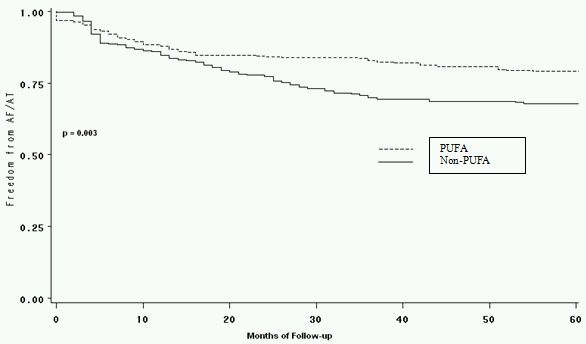
Kaplan Meier survival curve depicting the number of patients in each group that were arrhythmia free at the end of the follow-up period.

Thirty-five (27.1%) patients in the study group had early recurrence vs. 57 (44.1%) in the control group p-value< 0.0001. Twenty-nine (23.2%) patients in the PUFA group vs. 41 (31.7%) in the non-PUFA group had procedural failure (p-value < 0.003). Kaplan Meier curve was constructed to depict the number of patients that were arrhythmia free at the end of the follow up period (mean 28+/7 months) given in Figure 1.  There were no significant differences in complications between the PUFA and non-PUFA groups (Table 1).

## Discussion

This is the first study that assessed the benefit of treatment with PUFAs in patients that had undergone catheter ablation. The main findings of our study were: 1) Patients treated with PUFAs had lower incidences of early recurrence and procedural failure rates. 2) Patients treated with PUFAs had significantly lower pre and post CRP levels. 3) Patients treated with PUFAs had similar complication rates as the control population.

We observed that the anti-inflammatory properties of PUFAs appeared to be able to reduce the amount of inflammation thus, possibly lowering the incidence of early recurrence in patients that had undergone catheter ablation. Calo et al. conducted a randomized controlled trial that demonstrated that oral intake of PUFAs for at least 5 days prior to CABG reduced the incidence of postoperative AF by 54.4% [5]. While our success rates were not as dramatic as that reported by Calo et al., our study also demonstrated improvement in recurrence of AF postoperatively. Additionally, many of Calo's patients did not have a prior history of AF thus, they most likely did not have a complex pre-existing atrial substrate perpetuating AF.

We observed that patients on PUFAs had less procedural failure rates than the control group.  Mozaffarin et al. conducted a prospective, population-based cohort study of 4,815 adults who consumed tuna or other broiled or baked fish illustrating the association between PUFA consumption and lower incidences of AF [4].

Prior studies had reported that PUFAs consumption reduces incidence of strokes [7]. He et al in a prospective cohort study with 43,671 subjects showed that consuming 1 or more servings of fish reduce the risk of stroke in men during a 12 year follow-up [8].  Bouzan et al conducted a meta-analysis demonstrating that any fish consumption confers substantial relative risk reduction compared to no fish consumption  [9]. We observed no benefit in postprocedural stroke in our population.

## Limitations

While our study reported new findings, it was limited because it was a small retrospective study thus, had all the limitations inherent to a study of this design. However, all data were prospectively collected. We did not have information about patient dietary fish consumption; nevertheless, we have no reason to suspect that dietary intake was substantially different between subjects. We were not able compare the affects of variations in dosages because the groups would be too small for correct analysis.

## Conclusion

Patients treated with PUFAs had a lower incidence of early recurrence and procedural failure.

## Figures and Tables

**Table 1:**
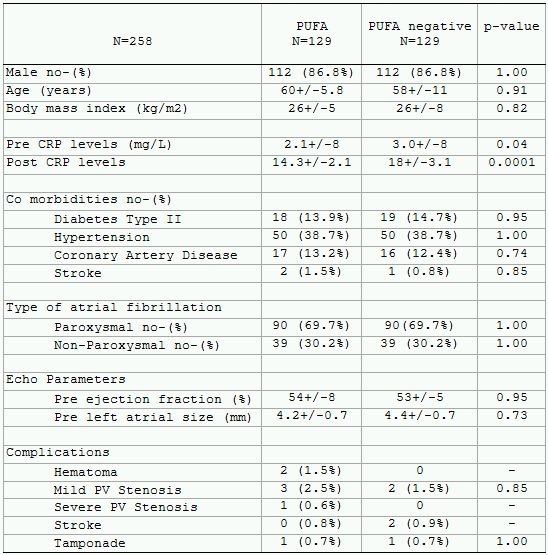
Demographics of the case controlled study and control populations
